# Hypo vs. hyperthyroid eye disease: is there any difference?

**DOI:** 10.1186/s12886-023-02806-7

**Published:** 2023-02-10

**Authors:** Bahram Eshraghi, Mohsen Pourazizi, Maryam Abbasi, Iman Mohammadbeigy

**Affiliations:** Isfahan Eye Research Center, Department of Ophthalmology, Isfahan University of Medical Sciences, Isfahan, Iran

**Keywords:** Thyroid-eye disease, Graves’ orbitopathy, Hypothyroidism, Hyperthyroidism

## Abstract

**Background:**

Thyroid-eye disease (TED) is the most common extra-thyroidal presentation of graves’ disease. We performed this study to compare clinical characteristics of TED in hypothyroid vs. hyperthyroid patients.

**Methods:**

This was a retrospective analytical cross-sectional study in which we compared demographics, severity (EUGOGO classification) and activity (clinical activity score) of TED, thyroid disease duration, TED duration and clinical signs between hypothyroid eye disease (Ho-TED) and hyperthyroid eye disease (Hr-TED). To minimize the effect of selection bias and potential confounders, 1:1 propensity score matching (PSM) was also performed.

**Results:**

Three hundred and seventy-four patients (341 Hr-TED and 33 Ho-TED) with a female to male ratio of 1.4:1 were identified in our study. Female to male ratio was 1.3:1 in hyperthyroid and 4.5:1 in hypothyroid group (*P* = 0.005). The duration of thyroid disease was longer in Ho-TED (*P* = 0.002) while the duration of eye disease was not significantly different between the Hr-TED (mean = 24.33 ± 41.69, median = 8) and Ho-TED (mean = 19.06 ± 33.60, median = 12) (P = 0.923). Most of the patients in hypothyroid group developed eye involvement after thyroid disease (80.0% in hypo vs. 48.1% in hyper, *P* = 0.003). Severity (*P* = 0.13) and activity (*P* = 0.11) was not different between Hr-TED and Ho-TED patients. After PSM analysis, no clinical characteristics were significantly different between the two groups (*P* > 0.05).

**Conclusion:**

The results of our study showed several differences between the Hr/Ho TED patients including sex, duration of thyroid disease and pattern of eye involvement. After matching the two groups with statistical methods, no clinical characteristics were different between Hr-TED and Ho-TED patients.

## Background

Thyroid-eye disease (TED) is the most common extra-thyroidal presentation of graves’ disease [1] which affects about 16/100000 women and 2.9/100000 men every year [2]. Although a rare disease, it is a therapeutic challenge in severe form and impairs quality of life even in mild form [3].

TED is most commonly seen with graves’ hyperthyroidism; however it can be associated with hypothyroidism or euthyroidism [4]. Globally, the prevalence of hyperthyroidism, hypothyroidism and euthyroidism in TED patients, is 86.2%, 10.36% and 7.9% respectively [5]. Ocular symptoms may appear before or after the thyroid manifestations but in 80% of cases they occur within 18 months of each other [6].

It is widely known that thyroid dysfunction, especially hyperthyroidism, relates to more severe form of TED and the treatment of thyroid dysfunction is a component of TED management [7]; However, there are far few studies that have compared clinical characteristics of TED with respect to primary thyroid function and they have opposing results as well. Kashkuli et al. [8] found that Ho-TED and Hr-TED patients have no significant differences in terms of disease severity, activity, clinical characteristics and demographics; however, in Ekcstein et al. study, euthyroid and hypothyroid TED patients showed less severity and activity and had more asymmetrical eye involvement [9]. In addition, Ponto et al. showed that euthyroid and hypothyroid TED, presents with more asymmetry and less severity [10].

There is scarcity of data about contrasting clinical characteristics of hypo, hyper and euthyroid TED patients [11–19]. In the present study we aimed to compare the clinical characteristics of hypo and hyperthyroid TED patients.

## Material and methods

### Study population and selection criteria

This was a retrospective analytical cross-sectional study. The participants were all consecutive Ho-TED and Hr-TED patients who referred to Feiz Eye Hospital (a tertiary referral center in Isfahan, Iran) and a private oculoplastic clinic over a period of 6 years, during 2015–2020.

Patients were assigned to the the hyperthyroid or hypothyroid group based on their thyroid function status before starting the treatment for hypothyroidism or hyperthyroidism. Patients with low thyroid-stimulating hormone (TSH) and normal or increased free thyroxine (T4) were considered as hyperthyroid and patients with increased TSH and normal or decreased free T4 were considered as hypothyroid.

The protocol of study was approved by the Ethics Committee of Isfahan University of Medical Sciences, Isfahan, Iran (IR.MUI.MED.REC.1399.1001).

The diagnosis of TED was made on the basis of 2014 American Academy Of Ophthalmology criteria for the diagnosis of TED [20].

### Data collection

The following data were extracted from patients’ medical documents and compared between the two groups: age, sex, severity and activity, thyroid disease duration, TED duration, the time interval between the onset of thyroid disease and eye involvement. TED severity was assessed according to European Group on Graves’ Orbitopathy (EUGOGO) guidelines classification [21]: mild TED refers to patients who had one or more of the followings: minor lid retraction (< 2 mm), mild soft-tissue involvement, exophthalmos < 3 mm above normal for race and gender, no or intermittent diplopia and corneal exposure responsive to lubricant. Moderate-to-severe TED refers to patients who have two or more of the followings: lid retraction ≥ 2 mm, moderate or severe soft tissue involvement or exophthalmos ≥ 3 mm above normal for race and gender, inconstatnt or constant diplopia. Sight-threatening TED are patients with dysthyroid optic neuropathy and/or corneal breakdown. TED activity was evaluated using seven points scale clinical activity score (CAS) and CAS ≥ 3 was considered as active TED [22]. The severity and activity of TED was assessed at the first time the patients presented at the oculoplastic clinic. In asymmetric cases, the worse score was considered for classification of activity and severity. All the patients were examined by one ophthalmologist to reduce inter and intra observer variability.

### Statistical analysis

Data analysis was performed using software SPSS (version 16, Chicago, IL, USA). Quantitative data were reported as mean ± SD and Median [min–max]. Qualitative data were reported as percentage and proportions. The normality of data, was assessed using Kolmogorov–Smirnov test. We used independent sample t-test to compare the means between the two groups, and Chi-Square or Fisher’s Exact Test (if the expected values were less than 5) to compare the nonparametric variables in the two groups. Non-parametric data were subsequently analyzed using the Mann–Whitney U test. A *p* value of < 0.05 was regarded as significant. Due to the low number of Ho-TED patients, 1:1 propensity score matching (PSM) was conducted to reduce the influence of selection bias and confounding effects. PSM baseline variables included sex and age. The nearest neighbor method was used to choose matched patients in hypo and hyperthyroid groups. *P*-values were obtained for each item after matching.

## Results

A total of 374 patients diagnosed with TED were identified in our study. The vast majority were hyperthyroid (341/374, 91.2%) and 33 patients were hypothyroid (33/374, 8.8%). The mean age of patients was 39.58 ± 13.53. Most of the patients (221/374, 59.1%) were women.

Comparison between hyper and hypothyroid TED patients is summarized in Table 1 (Table 1).

**Table 1:** Comparison between hyper and hypothyroid TED patients before PSM analysis

Variables	Hr-TED	Ho-TED	*P*-value
Sex, n (%)			
Male	147 (43.1%)	6 (18.2%)	0.005^*¶^
Female	194 (56.9%)	27 (81.8%)	
Age,			
Mean ± SD	39.45 ± 13.80	40.93 ± 9.94	0.405^Ω^
Median [min–max]	38 [10,72]	37.5 [21,65]	
Duration of thyroid disease (months)			
Mean ± SD	39.92 ± 55.75	61.33 ± 47.31	0.002^* Ω^
Median [min–max]	18 [1,300]	62 [2,180]	
Duration of eye involvement (months)			
Mean ± SD	24.33 ± 41.69	19.06 ± 33.60	0.923^Ω^
Median [min–max]	8 [1,288]	12 [1,180]	
Pattern of eye involvement, n (%)			
After thyroid disease	153 (48.1%)	24 (80.0%)	0.003^*¶^
Along With thyroid disease	114 (35.8%)	5 (16.7%)	
Before thyroid disease	51 (16.0%)	1 (3.3%)	
TED activity, n (%)			
Inactive (CAS < 3)	316 (92.7%)	28 (84.8%)	0.114^¶^
Active (CAS ≥ 3)	25 (7.3%)	5 (15.2%)	
TED severity, n (%)			
Mild	74 (23.4%)	9 (29%)	0.137^¶^
Moderate-to-severe	219 (69.3%)	17 (54.8%)	
Sight threatening	23 (7.3%)	5 (16.1%)	
Clinical sign, n (%)			
Presence of Corneal Ulcer	4 (1.2%)	0 (0.0%)	0.999^¶^
Presence of DON	19 (5.6%)	5 (15.2%)	0.032*^¶^
Presence of Restriction and Fixation of Globe	31 (9.1%)	2 (6.1%)	0.627^¶^
Presence of Eyelid Retraction	250 (78.6%)	19 (65.5%)	0.106^¶^

*Hr-TED* Hyperthyroid eye disease, *Ho-TED* Hypothyroid eye disease, *DON* Dysthyroid optic neuropathy, *CAS* Clinical activity score

^¶^Resulted from Chi-Square Test and fisher exact Test

^Ω^Resulted from independent Mann–Whitney U test

^*^Significance Correction is *P*-value < 0.05

Female/male ratio was 1.3:1 in hyperthyroid and 4.5:1 in hypothyroid group (*P* = 0.005). The duration of thyroid disease was longer in Ho-thyroid group (*P* = 0.002) while the duration of eye disease was not significantly different between the Hr-TED (mean = 24.33 ± 41.69, median = 8) and Ho-TED (mean = 19.06 ± 33.60, median = 12) group (*P* = 0.923). Most of the patients in hyperthyroid (153, 48.1%) and hypothyroid group (24, 80.0%) developed eye involvement after thyroid disease (*P* = 0.003) (Table 1).

Mean interval between thyroid and eye involvement in patients who developed eye involvement after thyroid disease was 30.63 ± 46.02 (median = 10) and 56.04 ± 38.04 (median = 59) months for Hr-TED and Ho-TED respectively. Mean interval between thyroid and eye involvement in patients who developed eye involvement before thyroid disease was 12.65 ± 24.96 (median = 3) and 3.00 (median = 3) for Hr-TED and Ho-TED respectively.

Active TED developed in 25 (25/341, 7.3%) of hyperthyroid and in 5 (5/33, 15.2%) of hypothyroid which reveals no significant difference (*P* = 0.11). The majority of patients had moderate-to-severe TED (Hr-TED 69.3% and Ho-TED 54.8%) or mild TED (Hr-TED 23.4% and Ho-TED 29%). There were no statistically significant differences between severity of Hr-TED and Ho-thyroid patients (*P* = 0.13).

Dysthyroid optic neuropathy (DON) developed in hypothyroid more than hyperthyroid group (19/341in Hr-TED vs. 5/33 in Ho-TED; *P* = 0.032). Corneal ulcer was seen in 1.2% of patients with hyperthyroidism (4/341in Hr-TED vs. 0/33 in Ho-TED; *P* = 0.999) (Table 1).

PSM was utilized to match 33 hyothyroid with 33 hyperthroid patients. Baseline demographic and clinical characteristics of the study groups after PSM analysis are presented in Table 2 (Table 2). After matching, no demographic and clinical characteristics were significantly different between the two groups (*P* > 0.05).

**Table 2:** Comparison between hyper and hypothyroid TED patients after PSM analysis

Variables	Hr-TED	Ho-TED	*P*-value
Sex, n (%)			
Male	6 (18.2%)	6 (18.2%)	1.000^¶^
Female	27 (81.8%)	27 (81.8%)	
Age,			
Mean ± SD	40.81 ± 9.73	40.93 ± 9.94	0.990 ^Ω^
Median [min–max]	42 [11,68]	37.5 [21,65]	
Duration of thyroid disease (months)			
Mean ± SD	57.16 ± 59.83	61.33 ± 47.30	0.757 ^Ω^
Median [min–max]	36 [1,120]	62 [2,180]	
Duration of eye involvement (months)			
Mean ± SD	42.96 ± 64.71	19.06 ± 33.60	0.094 ^Ω^
Median [min–max]	34 [1,50]	12 [1,180]	
Pattern of eye involvement, n (%)			
After thyroid disease	22 (68.8%)	24 (80.0%)	0.240^¶^
Along With thyroid disease	10 (31.3%)	5 (16.7%)	
Before thyroid disease	0 (0.0%)	1 (3.3%)	
TED activity, n (%)			
Inactive (CAS < 3)	31 (93.9%)	28 (84.8%)	0.427^¶^
Active (CAS ≥ 3)	2 (6.1%)	5 (15.2%)	
TED severity, n (%)			
Mild	8 (25.0%)	9 (29.0%)	0.185^¶^
Moderate-to-severe	23 (71.9%)	17 (54.8%)	
Sight threatening	1 (3.1%)	5 (16.1%)	
Clinical sign, n (%)			
Presence of Corneal Ulcer	0 (0.0%)	0 (0.0%)	-
Presence of DON	2 (6.1%)	5 (15.2%)	0.427^¶^
Presence of Restriction and Fixation of Globe	2 (6.1%)	2 (6.1%)	1.000^¶^
Presence of Eyelid Retraction	21 (70.0%)	19 (65.5%)	0.713^¶^

*Hr-TED* Hyperthyroid eye disease, *Ho-TED* Hypothyroid eye disease, *DON* Dysthyroid optic neuropathy, *CAS* Clinical activity score

^¶^Resulted from Chi-Square Test and fisher exact Test

^Ω^Resulted from independent Mann–Whitney U test

## Discussion

The present study, which includes 374 patients with TED, presents some difference between hyper and hypothyroid TED. These differences included sex, duration of thyroid disease, pattern of eye involvement, and presence of DON. However, none of these parameters reached statistically significant difference after PSM analysis. These findings can provide new insights into the clinical and epidemiological aspect of TED in hyper compared with hypothyroid patients.

Although TED is mostly seen with hyperthyroidism, it can occur with hypo or euthyroidism [23]. It is not completely understood how thyroid function affects TED phenotype [8, 9, 20]. In our study, hyperthyroid and hypothyroid patients accounted for about 91% and 9%, respectively. Our results were similar to a previous study performed in Iran that reported the prevalence of Hr-TED and Ho-TED 92.4% and 7.5% [8]; however, another study that was also conducted in our country, reported the prevalence of the Hr-TED and Ho-TED as 83.8% and 18.4% [12]. The difference may be due to its small sample size as its population was three times less than our study. As reported by a recent systematic review, globally, the prevalence of hyperthyroidism in TED is between 65.7 and 99.1%, hypothyroidism between 0.2% and 33.3% and euthyroidism between 0.9% and 15.4% [5]. These wide ranges may be associated with differences in ethnicity, genetics, environment and patient selection [5, 8, 10].

The sex distribution between Hr-TED and Ho-TED was significantly different in our study; however, like previous studies most of the patients in both groups were women. This is contrast to Kashkuli et al. [8], Ekcstein et al. [9], Leo et al. [24], Medghalchi et al. [12] findings that showed no difference between Hr-TED and Ho-TED regarding sex distribution.

Before PSM analysis, the number of TED patients with eye involvement before, along with or after thyroid disease was significantly different between Hr-TED and Ho-TED; however, no significant difference was observed after PSM. Most of the patients in two groups developed TED after thyroid involvement. Muralidhar et al. study [25] showed that most of Ho-TED, developed thyroid disease before TED and most of Hr-TED, developed thyroid disease after TED. In Ponto et al. study, most Hr-TED patients had thyroid disease before or simultaneously with TED. Our results before PSM analysis, are in contrast to Kashkuli et al. [8] study that found no difference between Ho-TED and Hr-TED regarding time interval between thyroid and eye disease. In Kashkuli et al. study, patient categorization was different from our study as patients were classified into three groups: Patients who developed eye disease within 18 months before or after thyroid disease, more than 18 months before thyroid disease and more than 18 months after thyroid disease and this may elucidate the different results.

In our study, Hr-TED and Ho-TED had the same severity and activity. This is in agreement with Kashkuli et al. [8], Leo et al. [24] and Ponto et al. [10] studies. Contrary to our study, Ekcstein et al. [9] found that euthyroid and hypothyroid patients present with less severe and active TED than hyperthyroid ones. Similarly, Muralidhar et al. [25] stated that euthyroid and hypothyroid eye disease have less severe disease. According to Rundle Curve diagram, TED in different times has different severity and Ekcstein et al. selected patients who presented 6 to 12 months from the onset of TED; but we did not have this inclusion criterion. It is noteworthy that in our study, duration of eye disease was similar in both groups and this shows that it did not confound our results. Differences in ethnicity, genetics, environment can also explain these discrepancies; as TED presents milder in Asia [26–29]. Different tools used for measuring severity may also clarify these inconsistencies.

Before and after PSM analysis, most of our patients were moderate to severe, then mild and ultimately sight threatening, respectively. Our findings are in keeping with Leo et al. study [24] and this may be due to the fact that both studies were conducted in a tertiary center where more severe cases are referred. Moreover, in developing countries like our country, timely referral in early stages does not occur and many patients are referred in advanced stages of the disease. In contrast to our results, most of the cases in Kashkuli et al. study [8], did not have severe TED. This may be explained by the fact that they selected their patients from an endocrinology clinic where there are more mild cases of TED that are not referred to eye clinics. In Muralidhar et al. [25] and Ponto et al. [10] studies, most hyperthyroid patients had moderate-to-severe and most hypothyroid patients had mild TED.

Before PSM analysis, we found that the prevalence of DON was higher in Ho-TED group; however, we did not find any statistically significant difference after PSM. In contrast to our study, Ekcstein et al. [9] concluded that none of the euthyroid and hypothyroid patients developed DON but 7% of hyperthyroid patients developed DON. In Ponto et al. [10] study, euthyroid and hypothyroid eye disease did not progress to sight-threatening form but 5% of Hr-TED had DON.

The mean age of our patients was about 40 years which was close to two other studies in our country [8, 12] and two other studies in southeast Asia, but less than what was reported in European Group on Graves’ Orbitopathy studies about 50 years [30, 31]. This may relate to younger population in our area. The mean age did not differ between Hr-TED and Ho-TED. Kashkuli et al. [8], Ekcstein et al. [9], Leo et al. [24] got the similar results.

Our study had some limitations. Low number of Ho-TED, restriction of the study to a tertiary referral center, lack of access to patients’ smoking and radioactive iodine treatment history, and lack of data about activity and severity of TED over time were limitations of our study. Lack of specific intervals to check thyroid status and long time to follow up were another limitation of our study. Although our study had some limitations, current study, which included 374 patients, could provide some references for the difference of Hr/Ho TED that there is limited data about this topic.

## Conclusion

There are some differences between the epidemiological features of Hr/Ho TED diseases; however, after PSM these features were not significantly different between the two groups. In the future, large-scale studies should be designed to evaluate Ho-TED to improve our understanding of this patient population.

## Data Availability

The datasets generated and/or analysed during the current study are not publicly available due to privacy and ethical restrictions but are available from the corresponding author on reasonable request.

## Abbreviations

TED: Thyroid-eye disease
Ho-TED: Hypothyroid eye disease
Hr-TED: Hyperthyroid eye disease,
EUGOGO: European Group On Graves’ Orbitopathy
CAS: Clinical activity score
DON: Dysthyroid optic neuropathy
PSM: Propensity score matching
